# Integrated Curriculum Enhancing Anatomical Publications at Geisinger Commonwealth School of Medicine

**DOI:** 10.7759/cureus.106321

**Published:** 2026-04-02

**Authors:** Mathangi Rajaram-Gilkes, Kelly Barr, Taylor Moglia

**Affiliations:** 1 Medical Education, Geisinger Commonwealth School of Medicine, Scranton, USA

**Keywords:** anatomical publications, clinical anatomy, flipped classroom, horizontal integration, integrated curriculum, medical education, physician mentors, research opportunities in medical school, vertical integration, virtual anatomy platform

## Abstract

Integrated medical curricula have gained increasing attention within anatomical sciences education for their potential to enhance early clinical reasoning and promote scholarly productivity. Since its implementation in 2022, the Integrated Medical Education Curriculum at the Geisinger Commonwealth School of Medicine (GCSOM), Pennsylvania, has demonstrated notable success, particularly in enabling first- and second-year students (2023-2025) to contribute to peer-reviewed publications in anatomical sciences. The curriculum incorporates horizontal and vertical integration of anatomy, physiology, pathology, pharmacology, and radiology, supported by experiential learning through cadaveric dissection, ultrasound-based anatomy, virtual microscopy, standardized patient encounters, and early physician collaboration. This study systematically reviews peer-reviewed publications authored by pre-clinical medical students enrolled in the integrated curriculum between 2023 and 2025. The analysis examines how interdisciplinary instruction, mentorship from anatomists and clinical faculty, and structured research opportunities empowered students to generate publishable anatomical and clinical observations. Individual curricular components were evaluated for their contributions to students’ skills in anatomical interpretation, clinical correlation, and scholarly dissemination. By the end of the second year, students demonstrated advanced integration of structural and functional anatomy with clinical findings. Their publications reflected competencies in identifying anatomical variants, correlating cadaveric findings with surgical procedures, and recognizing pathological conditions through imaging and dissection. These outcomes highlight the curriculum’s effectiveness in promoting early scholarly engagement. The GCSOM integrated curriculum significantly enhances anatomical comprehension and clinical correlation during the pre-clinical years. Through multidisciplinary mentorship and research-embedded learning, students acquire the analytical skills and confidence necessary to publish early in their medical training. These findings support integrated curricula as a powerful model for advancing anatomical scholarship among early-stage learners.

## Introduction and background

An integrated curriculum in medical education is an instructional approach that breaks down traditional subject silos (such as anatomy, physiology, and pathology) to blend the basic and clinical sciences, fostering a holistic understanding of health and disease. It merges disciplines around themes or organ systems, enabling students to apply theoretical knowledge to real-world patient scenarios. Implementation of this increased retention of knowledge, enhanced ability to apply concepts to clinical situations, and improved preparation for clinical work [1].

The Integrated Curriculum aims to dissolve the traditional separation between basic and clinical sciences by intentionally linking concepts across disciplines and across time. Its purpose is to strengthen learners’ understanding and retention by revisiting and applying knowledge in progressively complex ways. Key features include horizontal integration, which unites related subjects within the same phase of training, such as anatomy, physiology, and pathology of a specific system; vertical integration, which weaves foundational science with clinical experience throughout the entire curriculum; and contextualized learning, which uses real-world clinical cases to highlight the practical relevance of scientific principles early and consistently [2].

While institutions may have diverse missions, all accredited programs must demonstrate performance that meets the Liaison Committee on Medical Education (LCME) standards; local circumstances cannot justify substandard medical education [3]. LCME Standard 7 requires medical curricula to integrate biomedical, behavioral, and social sciences to support students’ mastery of modern medical practice. The curriculum must cover organ systems, the life cycle, prevention, symptoms and signs, differential diagnosis, treatment planning, and end‑of‑life care. It must also include instruction in scientific methods and in clinical and translational research - how these are conducted, evaluated, communicated to patients, and applied in care. Additionally, programs must intentionally develop students’ critical thinking and problem‑solving skills using evidence‑based approaches. In addition, LCME emphasizes preparing students to diagnose, prevent, report, and treat medical consequences of common societal problems [3].

The curriculum must train students to recognize and address personal, interpersonal, and systemic biases in health care. It should teach how cultural, social, and structural factors influence health beliefs and outcomes, and provide instruction in culturally and structurally competent care, the roots and impacts of health disparities, and strategies to reduce inequities. The curriculum also requires the development of strong communication skills for interacting with patients, families, and health professionals, and it prepares students for effective interprofessional collaboration through team‑based patient‑care experiences [3]. LCME Standard 8 requires schools to maintain a coherent, high‑quality curriculum through strong faculty oversight, defined program objectives, and ongoing evaluation. A central curriculum committee manages and reviews all curricular elements to ensure alignment with institutional goals and continuous improvement. Schools must use outcomes data and student feedback to guide revisions, ensure consistent instruction and assessment across sites, track completion of required clinical experiences, and uphold policies regulating student workload and required curricular time [3].

The following 12 tips have been suggested in 2011 to create an effective medical curriculum. They include effective training of faculty and staff; to decide on the scope of curriculum; choose the level of integration; incorporate both vertical and horizontal integration; establish working groups with respective responsibilities; determine learning outcomes; identify knowledge, skills and attitude; create themes; prepare an effective timetable; select assessment methods; establish good communication with staff and students, and re-evaluate and revise when necessary [4].

Horizontal integration is defined to be an integration between parallel disciplines, such as anatomy, physiology and biochemistry or medicine, surgery and therapeutics traditionally taught in the same phase of the curriculum and vertical integration as an integration between disciplines traditionally taught in different phases of the curriculum. It can occur throughout the curriculum, with the basic medical and clinical sciences beginning together in the early years of the curriculum and continuing until the later years [4].

Medical education has evolved over the past two decades in response to rapid scientific advances and new medical technologies, prompting a shift toward more effective ways of teaching core concepts. Emerging curricular trends emphasize conceptual rather than detail-heavy learning, integrated rather than siloed subject instruction, the use of electives to complement required coursework, and small-group, problem-based learning that engages students in clinical reasoning. The likely future model blends traditional teaching with concept-driven, integrated curricula and case-based small-group learning using both real and hypothetical clinical scenarios [5].

Vertical integration is more than a curricular structure - it is an educational philosophy that shapes learners’ professional development and engagement. It extends across the entire continuum of medical training, supporting lifelong learning. The updated definition highlights this to be a progressive process in which learners increasingly participate in the professional community through growing, knowledge-based involvement and gradually expanding responsibilities in patient care [6].

**Figure 1:**
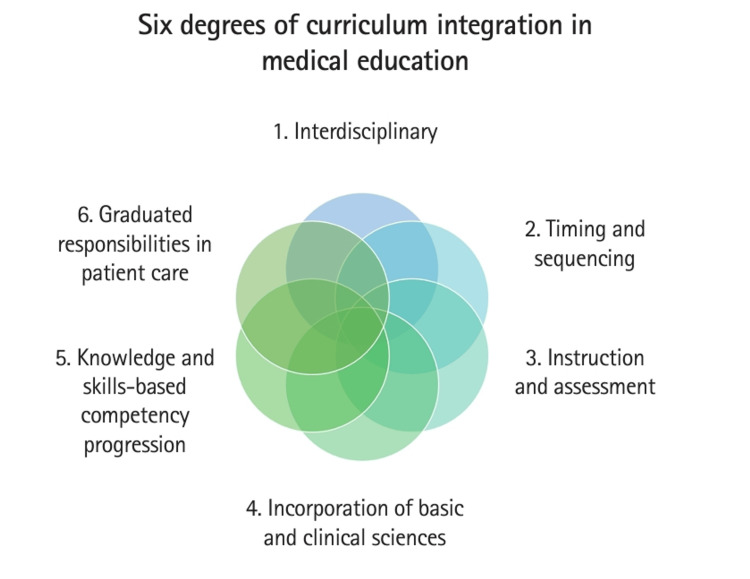
The six degrees of curriculum integration in medical education in the United States. Figure adapted from Youm et al. [7], published under the terms of the Creative Commons Attribution License (http://creativecommons.org/licenses/by/4.0/).

Another new model presents six degrees of curriculum integration, incorporating the following concepts (Figure 1) [7]: (1) interdisciplinary, (2) timing and sequencing, (3) instruction and assessment, (4) incorporation of basic and clinical sciences, (5) knowledge and skills-based competency progression, and (6) graduated responsibilities in patient care [7].

The condensed essence of the six degrees of curriculum integration involves interdisciplinary integration, which combines two or more disciplines into shared curricular segments. Timing and sequencing structures when and in what order content is presented across courses, phases, or sessions. This ensures learners encounter integrated material at appropriate times across their program, not just within single blocks. Instruction and assessment use teaching and assessment methods such as problem-based learning, team-based learning, and simulation that actively support integrated learning. This degree also prioritizes formative assessment, feedback, and reflective practices that capture applied, not just factual knowledge [7]. Intentional integration of basic and clinical sciences mirrors how physicians think in practice - drawing on both simultaneously - and fosters holistic clinical reasoning. Graduated responsibilities in patient care ensure learners increasingly participate in patient care within a system-based context. This draws on health systems science, emphasizing how healthcare delivery and interprofessional collaboration work, and how clinical thinking shapes competent, adaptable physicians [7]. The above concepts are thoroughly integrated and delivered in the medical curriculum at Geisinger Commonwealth School of Medicine (GCSOM).

The mission statement of GCSOM is focused on educating aspiring physicians and scientists to serve society using a community-based, patient-centered, interprofessional, and evidence-based model of education that is committed to fairness and respect for all, promoting discovery and utilizing innovative techniques. The Total Health Curriculum (THC) adopted by GCSOM develops skilled, compassionate, and adaptable physicians through a learning-science-based approach that emphasizes individualized patient care, community health, and personal growth. Within three phases, it covers preclinical foundations in biomedical science and clinical reasoning, early clerkship immersion across major disciplines, and a flexible, career-focused final phase with electives and advanced rotations, respectively. Key features include a clinical presentation model, flipped-classroom learning, and longitudinal Systems, Society, and Humanism in Medicine (SSHM) themes, which promote preventive primary care, population health awareness, health access and equity, community engagement, system-based practice, and professional identity formation, as suggested by LCME [3]. Educational objectives span patient-centered care, professionalism, health system science, critical thinking, and clinical skills. A clinical skills and simulation center helps students to practice interviewing, communication, examination, and procedures through standardized patients, high-fidelity simulators, a Sectra 3D anatomy table, and a range of task trainers.

The research and scholarship office supports student and faculty research, compliance, and scholarly growth across the THC. Students can engage in an eight‑week Phase 1 summer period through research, clinical internships, service learning, or the Summer Research Immersion Program (SRIP), and students complete the Collaborative Institutional Training Initiative (CITI) for research ethics. Long‑term options include the Medical Research Honors Program (MRHP), featuring a hypothesis‑driven project, thesis, and defense, and other research opportunities. Several opportunities are made available to students so they may pursue community service and global health experiences and apply for enrichment funding. The above being the foundation offered throughout all the phases, the scholarly work done by students engaged in this curriculum is hereby reviewed to highlight the salient aspects that enabled the dissemination of such.

## Review

Results

This is a narrative review of nine peer-reviewed publications authored by 18 pre-clinical medical students and three faculty mentors enrolled in the integrated curriculum between 2023 and 2025. The article examines how interdisciplinary instruction, mentorship from anatomists and clinical faculty, and structured research opportunities empowered students to generate publishable anatomical and clinical observations. Based on the LCME-compliant educational competencies [3] adopted by GCSOM, students are exposed to various educational platforms and teaching modalities that provide opportunities to do research, mainly due to the horizontal and vertical integration implemented during Phase 1 of the curriculum. The following salient components of this curriculum at GCSOM have been identified as playing a crucial role in the successful dissemination of their scholarly work. Those components include (1) foundational sciences and early systems integration, (2) advanced systems and clinical integration, (3) cadaveric dissection and anatomy lab experience, (4) virtual anatomy and imaging resources, (5) pathology and hospital-based learning, (6) case-based learning and clinical skills development, (7) clinical observations in lab leading to research ideas, (8) team-based learning, (9) physician and patient-centered care (PPCC), and (10) collaborative opportunities.

Discussion

Foundational Sciences and Early Systems Integration

Phase 1 of the integrated curriculum is organized into clinical presentation modules, covered over 18 months in various blocks, including foundational concepts, musculoskeletal, gastrointestinal, cardiology, pulmonary, renal, hematology, endocrine, reproduction, and neurosciences. During these integrated sciences blocks, core disciplines such as anatomy, embryology, physiology, genetics, cell biology, microbiology, histology, pharmacology, and pathology are delivered along with clinical sciences to establish horizontal and vertical integration. The clinicians’ presence and sharing of clinically relevant information help immensely with real-life scenarios and clinical outcomes that the students will encounter in their clinical space. Such early instruction in neuroanatomy, vascular anatomy, and pathology allowed students to recognize and write thoughtful discussions on how aberrant muscular origins, such as the high humeral origin of the pronator teres, can alter neurovascular pathways and produce clinically meaningful consequences [8]. Curricular clinical correlation through case-based learning and clinician participation emphasized the impact anatomical variation has on diagnosis, procedural planning, and surgical risk [8]. Some articles reflect on the clinical impact of implications for arteriovenous fistula creation, fracture management, and differentiation of median nerve compression syndromes [9]. Early systems integration also built a strong framework (especially histology and its clinical significance) for students to interpret the tissue composition of the urethrovaginal septum, including collagen subtypes, elastin distribution, and neurovascular structures. Understanding of these “basics” allowed for a more robust discussion of potential clinical implications based on histologic findings [10].

Advanced Systems and Clinical Integration

The systems-based pre-clinical curriculum at GCSOM allows for early integration of anatomy education with pathological changes and clinical manifestations. This allows students to build upon a strong foundation and apply anatomical concepts to clinical scenarios, a tenet crucial for all physicians and especially surgeons. It not only helps students to directly visualize and palpate structures in the lab but also helps focus on regional anatomy in the virtual simulation. The opportunity to explore typical anatomy virtually and in the cadaver lab provides a profound foundational understanding of anatomical structures. This helps in the identification of variants and clinical procedures done to improve the longevity of an individual [11]. Incorporating anatomy education and clinical integration early on in a student’s career highlights the importance and relevance of a strong foundational anatomy education, allowing students to acknowledge the benefits and potential drawbacks of the lack of such [12]. Literature reviews not only served as learning opportunities for anatomical variants but also helped correlate key clinical concepts associated with point-of-care ultrasound findings, which was emphasized in the musculoskeletal block [13].

Cadaveric Dissection and Anatomy Lab Experience

The lab experience involved cadaveric dissection in groups of 4-5 students to one cadaver, emphasized by post-session formative assessments. This hands-on experience strengthens anatomical knowledge and fosters respect for body donors who have willfully donated their bodies and who almost serve as the first patient for our students. This provides students with the opportunity to connect underlying normal anatomical structures with abnormal variations and pathological findings, and to correlate these with clinical relevance. By building upon a strong foundational knowledge base, students were able to recognize deviations from typical anatomy and appreciate their potential clinical significance. A central factor enabling these discoveries is the gross anatomy laboratory, where small-group cadaveric dissection promotes careful observation, application of knowledge, peer-to-peer discussion, and development of tactile skills [8,9,11].

Virtual Anatomy and Imaging Resources

Virtual anatomy instruction involves the utilization of the Sectra table. This interactive virtual high-definition 3D imaging platform displays anatomical structures from CT/MRI datasets, enabling a three-dimensional approach to gross and microscopic structures. This learning platform supports a review of anatomical structures, promotes team-based learning, and provides opportunities for case-based teaching. Students can virtually dissect, segment, and manipulate anatomical layers and visceral organs. Virtual microscopic images of histology and histopathology are integrated within this platform, which helps in the horizontal integration of basic sciences. The curriculum provides clinical vignettes that integrate the gross images with those of histology slides, X-rays, CT scans, MRIs, and ultrasound. Such assessments, integrated with clinical input from clinical experts, provide a solid foundation in phase one. This curricular component promoted student attention to details such as muscle origin and insertion points, leading to successful projects. Deliberate integration of foundational sciences, cadaveric dissection, and pathology-driven learning with access to virtual anatomy and imaging resources provided students with the imaging background and necessary resources to interpret the clinical significance of these findings [14].

Pathology and Hospital-Based Learning

During lab sessions, students are exposed to pathological specimens brought by visiting pathologists associated with Geisinger hospitals. A visit with collaborating pathologists at the local hospital provides students with a thorough idea of procedures done within a hematology lab, routine histological slide preparation, and visualization of light and electron microscopy and allows participation in cryosectioning. Hands-on involvement in one such project involved the use of four different stains to study the composition of the urethrovaginal septum, an area in the human body that had rarely been published in the literature. Virtual images prepared from sections within this region with hematoxylin, eosin, trichrome, and silver stains were compared and published with relevant clinical significance [10]. This mentorship also helped bridge the gap for students between basic science and clinical significance, giving students guidance in recognizing how microanatomical findings might translate into the pathophysiology of conditions such as pelvic organ prolapse or stress incontinence [10].

Case-Based Learning and Clinical Skills Development

Students practice with standardized patients and discuss clinical vignettes covering pathology, management, prevention, and psychosocial and medico-legal issues. This prepares them for clinical rotations in Phase 2 with strong clinical reasoning and communication skills. This has provided the foundations for publications regarding PoCUS, radiological findings in covid patients, and the effects of a lack of medical knowledge resulting in iatrogenic causes of diseases or death [10,12,14].

Clinical Observations in Lab Leading to Research Ideas

Students observe a spectrum of clinical procedures as part of the integrated curriculum - from minor cyst excision to coronary artery bypass graft (CABG) and joint replacements - helping them correlate the significance of normal human anatomy. The discovery of CABG, a replaced hip or knee joint, inguinal hernia repair, an abdominal or cerebral aneurysm, or any other graft within cadavers sparks research interests. In addition to this, physiological variation in muscle origin or the course of neurovascular structures through the body also inspires them to look into clinical relevance. Such curiosity has resulted in publications on abdominal aneurysm with femoro-femoral bypass, cerebral aneurysm observation during a bifrontal craniectomy done by the neurosurgery team, and identification of the high origin of the pronator teres and abnormal bifurcation and course of the median nerve and brachial artery bilaterally [8,9,11,15].

Team-Based Learning

The sessions, called workshops, held twice weekly, involve small-group problem solving using board-style clinical vignettes. Students collaborate, teach peers, and use available resources to research answers. Clinicians with content expertise from varied specialties, such as gastroenterologists, orthopedicians, cardiologists, pulmonologists, nephrologists, hematologists, etc., participate in connecting content to clinical practice and strengthening teamwork, communication, and reasoning skills. The workshop sessions also reinforce the collaborative synthesis of new information and peer-to-peer teaching. With multiple students working together on various projects, the teamwork and collaborative peer teaching were profound in certain publications involving PoCUS, radiological findings in COVID cases, histological studies involving the urethrovaginal septum, and iatrogenic causes of human morbidity and mortality [10,12-14].

PPCC

PPCC integrates competencies such as medical knowledge, patient care, professionalism, communication, and systems-based practice. Students develop interviewing, clinical reasoning, and physical exam skills through structured modules and clinical exams. Emphasis on reflection, humanism, and cultural awareness supports professional identity formation and prepares students to access patient health care records via Epic, an electronic health record (EHR) software [13], and for community-centered healthcare. The weekly integrative sessions provided a strong foundation for formulating the discussion of papers, along with reflection, empathy and respect for donor bodies, and professionalism [8-11,15].

Collaborative Opportunities

During summers and integration weeks, students collaborate with nearby institutions such as Marywood University and the University of Scranton in Pennsylvania. Joint cadaveric dissections have led to the identification of certain anatomical muscle variants, which are currently being researched and are in various stages of development for publication. Collaborative opportunities with external institutions, such as Tulane University School of Medicine, have resulted in publications through faculty participation [16]. These partnerships strongly expand anatomical understanding, research skills, and inter-professional communication skills.

Table 1 indicates the names of authors involved in the relevant publications, a brief note on the article content, and the key components of the integrated curriculum that enhanced scholarly dissemination.

**Table 1:** Authors involved in the relevant publications, a brief note on the article content, and the key components of the integrated curriculum that enhanced scholarly dissemination. GCSOM: Geisinger Commonwealth School of Medicine

Authors From GCSOM	Key Component of Integrated Curriculum
Rajaram-Gilkes M, Fung K, Kiniale C, Adams W (2024) [8]	The article deals with bilateral variations in the pronator teres muscle. Foundational sciences and early systems integration: Cadaveric Dissection and Anatomy Lab Experience: Clinical Observations in Lab Leading to Research Ideas: PPCC.
Rajaram-Gilkes M, Burwell J, Barr K, Marcincavage D, Fung K, Chuwuemeka E (2023) [9]	The article covers bilateral variations in the median nerve and the brachial artery. Foundational sciences and early systems integration: Cadaveric Dissection and Anatomy Lab Experience: Clinical Observations in Lab Leading to Research Ideas: PPCC.
Rieker F G, Rajaram-Gilkes M, Barr K, et al. (2024) [10]	The article encompasses a histological study of the urethra-vaginal septum. Foundational sciences and early systems integration. Pathology and Hospital-Based Learning: Case-Based Learning and Clinical Skills Development: Team-Based Learning. PPCC.
Fung K, Rajaram-Gilkes M, Moglia T, Rieker F, Falkenstein C (2024) [11]	The article is a case study of a large abdominal aneurysm, with multiple repairs and a femoro-femoral bypass graft. Advanced Systems and Clinical Integration: Cadaveric Dissection and Anatomy Lab Experience: Clinical Observations in Lab Leading to Research Ideas: PPCC.
Moglia T, Falkenstein C, Rieker F, Tun N, Rajaram-Gilkes M (2024) [12]	The article addresses human morbidity and mortality secondary to ignorance of anatomical landmarks and their clinical significance. Advanced Systems and Clinical Integration: Case-Based Learning and Clinical Skills Development: Team-based learning.
Adams W, Chukwuemeka E, Kiniale C, Bekker J, Johnson H, Rajaram-Gilkes M (2024) [13]	The article covers the PoCUS implementation within the Geisinger system. Advanced Systems and Clinical Integration: Team-based Learning: Data visualization, analytical thinking, and clinical application: Access to the health care system and analysis of the data: PPCC.
Rajaram-Gilkes M, Shariff H, Adamski N, Costan S, Taglieri M (2023) [14]	This is a review article on crucial radiological investigations in COVID management. Virtual Anatomy and Imaging Resources: Case-Based Learning and Clinical Skills Development: Team-based Learning.
Burwell J, Rajaram-Gilkes M (2024) [15]	This article discusses a new technique—bifrontal craniectomy—to decompress intracranial fossae during traumatic conditions. Clinical Observations in Lab Leading to Research Ideas: PPCC.
Rajaram-Gilkes M (2023) [16]	The article is an anatomical study of landmarks in the skull, facilitating the approach to the posterior cranial fossa during surgery. Collaborative opportunities. Collaboration with external institutions. Cadaver dissections, data visualization, analytical thinking, and clinical applications.

Limitations of scholarly activities during the pre-clinical phase of study

Scholarly engagement is an important component of a strong residency application, particularly for competitive specialties. This pressure often motivates pre-clinical medical students to devote substantial time to research at the expense of focused academic study. In addition to the significant time commitment, many projects require students to commute to hospital-based settings or collaborate across institutions, which introduces added financial and logistical burdens. Certain types of scholarly work, such as anatomy‑based research, demand meticulous attention, further extending the required time for dissections, high‑quality imaging, and careful labeling of cadaveric photographs. Literature review projects can also consume large portions of a student’s schedule as they navigate extensive prior work and synthesize complex findings.

Projects involving human subjects add another layer of complexity, as accessing clinical data typically requires IRB approval. Navigating these regulatory processes often demands additional initiative and persistence from students who are still learning how to engage with healthcare systems. Collaboration with clinical content experts may also be challenging, requiring ongoing communication, patience, and flexibility to align schedules and expectations. Once a manuscript is submitted, the review and revision process introduces further delays and requires continued effort in responding to feedback from editors and peer reviewers. Although students may have opportunities to present their work at regional or national conferences, participation is frequently limited by financial constraints and mandatory in‑class activities that restrict travel. Collectively, these factors highlight the substantial barriers that pre‑clinical medical students face when attempting to engage deeply in scholarly work, despite the clear value such experiences hold for their professional development.

## Conclusions

With a well‑timed, carefully sequenced interdisciplinary approach - and with exemplary instructional planning and assessment design - the curriculum at GCSOM achieves strong horizontal and vertical integration that enhances students’ knowledge and competency‑based skills. This integrated model has empowered students to engage meaningfully in research and scholarly activity in the field of anatomical sciences from the very beginning of their medical education. During the pre-clinical years, students not only gain a solid foundation in anatomy and clinical application but also develop advanced abilities in data visualization, scientific communication, and analytical thinking. In our view, the early success of students in producing scholarly work and achieving publication would not be possible without the knowledge, skills, and structured research opportunities afforded by the integrated medical curriculum. These elements collectively promote student empowerment and foster academic productivity at an early stage of their medical careers.
